# Cognitive Deficits in Female GPR158 Knockout Mice Revealed by Automated Home‐Cage Testing

**DOI:** 10.1111/gbb.70066

**Published:** 2026-08-18

**Authors:** Alyson P. Blount, Shradha V. Darira, Emmanuelle B. Palmieri, Laurie P. Sutton

**Affiliations:** ^1^ Department of Biological Sciences University of Maryland Baltimore County Baltimore Maryland USA

**Keywords:** cognition, GPCR, GPR158, spatial learning

## Abstract

GPR158 is a G protein coupled receptor (GPCR) that is abundantly expressed in the brain and has been shown to organize synaptic architecture and regulate synaptic function. Previous studies have demonstrated a role for GPR158 in hippocampal‐dependent learning and stress‐related affective behaviors yet its contribution to cognitive function under naturalistic, low‐stress conditions remains unclear. Here, we investigated the role of GPR158 in spatial learning, cognitive flexibility, and working memory using both traditional and automated home‐cage paradigms. The use of automated, socially enriched testing paradigms minimizes stress‐related confounds and allows us to discover the naturalistic roles of GPR158 in cognition and behavior. Using the IntelliCage to test spatial learning and working memory in a social enriched environment, we found that female GPR158 knockout mice exhibited deficits in spatial learning but showed intact working memory. Consistent with these findings, spatial impairments were also observed in the Barnes Maze, where female GPR158 KO mice showed pronounced deficits in spatial strategy acquisition, increased latency, reduced time spent in the reward area, and impaired performance during the probe trial. Male GPR158 knockout mice showed no significant difference in either spatial learning or working memory compared to their wild‐type littermates in the IntelliCage, whereas in the Barnes Maze knockout mice showed mild impairment in spatial strategy acquisition. These findings reveal a profound impairment with female GPR158 knockout mice in hippocampal‐dependent cognition and highlight the importance of low‐stress, ethologically testing environments.

## Graphical abstract

This study examined the role of GPR158 in cognitive function using both traditional and automated home‐cage paradigms that allow for testing in a social environment. Results showed that female GPR158 showed impairment in spatial learning and cognitive flexibility.

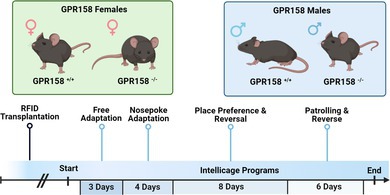

## 1 Introduction

GPR158 is a G protein coupled receptor (GPCR) that has been heavily implicated in cognition and affective states. GPR158 is found highly expressed in the central nervous system (CNS), including in the prefrontal cortex, nucleus accumbens, and hippocampus [1, 2, 3]. With its high expression in the brain, GPR158 has been shown to mediate synaptic transmission, neuronal excitability, and structural plasticity [4, 5]. This has been closely examined within the hippocampus where GPR158 acts as a specific synaptic organizer within hippocampal neurocircuits [6]. In the CA3 region, GPR158 plays a role in establishing synaptic functioning of developing mossy fiber‐CA3 synapses. It was shown that the loss of GPR158 not only disrupts morphology but also reduces synaptic strength at this synapse. GPR158 expression in the CA1 pyramidal neurons has been shown to regulate intrinsic excitability, and decreases in GPR158 levels disrupt dendritic architecture leading to reduced dendritic length and branching complexity [7]. GPR158 plays a role in spatial learning as mice deficient in GPR158 have been shown to display impairments in the acquisition and retrieval of spatial memory in the Morris Water Maze (MWM) test and show deficits in the novel object recognition task (NOR) [7, 8] As a major component for establishing hippocampal connections, GPR158 may also play a role in other hippocampal‐dependent learning tasks.

Studies have also implicated GPR158 signaling in stress‐induced depression, and maintenance of social interaction [8, 9, 10]. GPR158 levels are directly regulated by stress exposure via a corticosteroid‐mediated mechanism due to the presence of glucocorticoid response elements in the promoter region of GPR158 [11]. Mice subjected to chronic stress paradigms such as unpredictable chronic mild stress (UCMS) and physical resistant stress (PRS) exhibit an upregulation of GPR158 levels in the prefrontal cortex [3]. Elevated GPR158 levels have also been observed in the hippocampus after the stress of an immediate shock [12]. Increased GPR158 levels have been shown to influence various behavioral responses including depressive‐like responses in the tail suspension test and forced swim test, whereas GPR158 knockout (KO) mice display antidepressive and anxiolytic behaviors [3].

Stress exposure can lead to both adaptive and maladaptive responses and can affect cognitive performance based on the extent and duration of the stressor. Many cognitive tests for rodents induce stress intrinsically from the test itself or extrinsically from experimenter intervention such as repetitive experimenter handling of mice, new environmental factors, testing during rest period, and single‐housed animals [13, 14]. In addition, traditional behavioral testing often necessitates the isolation of individuals, which has been shown to decrease spatial learning, synaptic plasticity, and hippocampal neurogenesis [15]. Social interaction in a group setting has been found to alleviate stress‐induced cognitive and memory impairments in response to restraint stress and fear conditioning [16, 17]. Given the direct involvement of GPR158 in stress responses, it is unclear if this effect influences learning and memory processes.

Given the implication of GPR158 in cognitive processing, this study will further characterize GPR158 knockout mice in a series of learning and memory paradigms. As stress exposure and sociability can affect cognitive ability, we opted to use an automated home cage that monitors group‐housed behaviors. Testing within a home cage environment in a group setting has been achievable as each mouse is implanted with a radio frequency identification (RFID) and certain activities can be continuously monitored long‐term. In this report, we examine the effect of GPR158 on spatial learning and memory, cognitive flexibility, and working memory in a longitudinal, social, and minimally stress‐inducing environment to assess a more naturalized role of GPR158 in cognition.

## 2 Materials and Methods

### 2.1 Animals

GPR158 knockout (*GPR158* ^*−/−*^) mice have been previously described and originally purchased from the KOMP (GPR158^tm1(KOMP)Vlcg^). *GPR158* ^*−/−*^ mice were maintained on a C57/Bl6 background and bred as heterozygous mating to generate *GPR158* ^*−/−*^ and *GPR158* ^*+/+*^ wildtype littermates. Animals were group housed under a 12/12‐h light/dark rhythm, where food and water were provided *ad libitum*. All mice were group housed per sex after weaning and before being placed in the IntelliCage. Both male and female mice were used between the ages of 2–5 months. All experiments were conducted and granted approval in accordance with the Institutional Animal Care and Use Committee regulations at the University of Maryland Baltimore County.

### 2.2 Barnes Maze

Barnes maze was conducted similar to the procedure previously described [18, 19]. The maze consisted of a 92 cm circular white platform with 20 equally spaced holes (5 cm in diameter, 5 cm from the edge) 95 cm above the ground [20]. A black acrylic box could be fitted under any of the holes as the escape tunnel. The apparatus was stationed in the middle of the testing room and was lit by bright fluorescent lights on the ceiling. The illumination level was approximately 900 lx. A variation of fixed visual cues was positioned around the apparatus. The paradigm was conducted with white noise and cold air blown onto the platform. All trials were recorded using an overhead camera and were analyzed using ANYMAZE tracking software. The Barnes maze protocol consists of 5 days and three stages: habituation, acquisition training, and probe trial. Mice were habituated to the holding room for 30 min before starting each stage. During the habituation stage, mice were placed in the escape tunnel for 1 min before they were moved to the center of the platform and allowed to explore for 300 s or until they find the escape tunnel. Acquisition training was conducted for 4 days with 4 trials per day. During acquisition, mice were given 180 s to find the escape tunnel. If not found in the 180 s, mice were led to the escape tunnel. The latency to find the escape tunnel, distance, spatial strategies, and the percent (%) time spent in the reward area (the area immediately around the escape tunnel) were evaluated. The probe trial was used to assess the type of strategy used to find the escape tunnel. During the probe trial, the escape tunnel was removed, and mice were tested for 60 s. The time spent in the reward quarter was assessed. Spatial strategies were evaluated based on categories of search strategies [21]. The strategies used to find the escape tunnel were assessed during the acquisition period of the Barnes Maze and were scored blindly. Eight possible search strategies were differentiated into Spatial or Non‐spatial strategies. Non‐spatial strategies were thigmotaxis (path limited to the edges), random search (no spatial search pattern), scanning (random strategy that avoids the sides), and chaining (spatially non‐specific strategy where mice search in a fixed distance from the edges). The spatial strategies consist of indirect search (spatially targeted search with major directional error), focal search (spatially restricted search), directed search (slight deviation from the direct path), and direct path (nearly perfect trajectory with minimal deviation). The cognitive performance was evaluated using a scoring system with each of the 8 possible strategies being assigned a score so that the higher the cognitive strategies, the higher the score. Scoring numbers were assigned as follows: thigmotaxis = 0, random search = 1, scanning = 2, chaining = 3, indirect and focal search = 4, directed search = 5, and direct path = 6. The strategies were assessed for each trial per mouse, averaged per day, and normalized to 6, the highest possible score.

### 2.3 IntelliCage System

The IntelliCage system (https://www.tse‐systems.com/) is a fully automated home cage that is equipped with four operant conditioning chambers in each corner. Each corner has 2 drinking bottles and access is restricted to one mouse at a time by motorized doors that can be programmed based on mouse identification, time, and conditioned actions. Mice were identified by radio‐frequency identification (RFID) transponders to allow for individual identification at the entrance of a conditioning corner and the number/duration of visits, nose‐pokes, and licks were recorded. Mice can enter the corner through an opening containing a transponder reader antenna and their presence is confirmed by a temperature sensor which indicates a visit. A nose‐poke is characterized by the mouse inserting its nose into the opening to the waterspouts regardless of whether the door is opened and is registered by a light beam sensor. Licks are registered by a lickometer and are counted anytime the mouse touches the drinking spout. IntelliCage software 2.1 was used to program and monitor behavior as previously described [22, 23]. Standard chow was provided *ad libitum*.

### 2.4 Free Adaptation

Mice were separated into groups with both GPR158^+/+^ and GPR158^−/−^ mice and placed into 2 IntelliCages. During the first 3 days, all four corners were opened allowing full access to 2 water bottles in each corner. Visits, nose‐pokes, and licks were measured during the day and night cycles. Latency to the first response at any of the corners was measured and quantified.

### 2.5 Nose‐Poke Adaptation

Nose‐poke Adaptation consisted of 4 days directly after the conclusion of Free Adaptation. All doors were closed and could be opened once per visit to give access to the lickometer for 7 s before closing.

### 2.6 Place Preference and Reversal

The doors to all corners are closed and access to water was restricted to the two least preferred corners based on preference in Nose‐poke Adaptation. For Place Reversal, the correct corners were switched to the incorrect corners in Place Preference. The two correct corners were across from each other. A visit and a nose‐poke at the correct corner were required for the door to open, allowing access to water for 7 s. Visits and nose‐pokes at incorrect corners were registered but not rewarded with water access. The lick number, place error, and the proportion of corner return were measured. The total lick number was also measured as mice were only able to lick at correct corners. The Place Error was calculated as the number of incorrect initial nose‐pokes by total responses and was represented as percent (%) incorrect. Proportion of Corner Return was defined by 2 consecutive visits as follows: Correct to Incorrect (C–I) corner visits, Correct to Correct (C–C) corner visits, and Incorrect to Incorrect (I–I) corner visits. The percent use of pattern of return was calculated by dividing the number of each category of return by all pattern use. The delta (Δ) change in response was determined by calculating the change in measured parameters between the first day (initial) and the last day (final) of Place Preference, Place Reversal, or Patrolling.

### 2.7 Patrolling

All corners were changed to be incorrect corners except the reward corner. On a correct visit, nose‐poke, and lick, the reward corner was inactivated and switched in a clockwise direction to the next corner. A correct visit was cued with a green LED light. The mice had to learn to patrol to find the reward corner using visual discrimination and working memory.

### 2.8 Statistical Analysis

Statistical analysis was performed using GraphPad Prism 11 or R software. Barnes Maze cognitive performances were analyzed using an aligned‐rank transform (ART) approach implemented in the ARTool package for R. Sex, genotype, and training day were included as fixed effects, and mouse genotype was included as a random effect to account for repeated observations from the same animals across training trials. Probe trials of the Barnes Maze used a two‐way analysis of variance (ANOVA) model using heteroscedasticity‐consistent HC3 estimators to account for unequal variances and unbalanced sample sizes. For IntelliCage experiments, visits, licks, nose‐pokes, and latency were compared using a three‐way ANOVA with sex and genotype as between‐subject factors and day as a repeated‐measures factor. Where appropriate, a two‐way AVONA with repeated measures was used to assess genotype across training days. Response categories (C–C, C–I, I–I) in the preference, reversal, and patrolling experiments were analyzed using an ART approach where each mouse contributed to three response categories measured on Day 1 (initial phase) and Day 4 (final phase) and included phase and its interaction with genotype, sex, and response while accounting for repeated responses from each mouse. Statistical significance was set at *p* < 0.05. Data are represented as mean ± SEM.

## 3 Results

**FIGURE 1:**
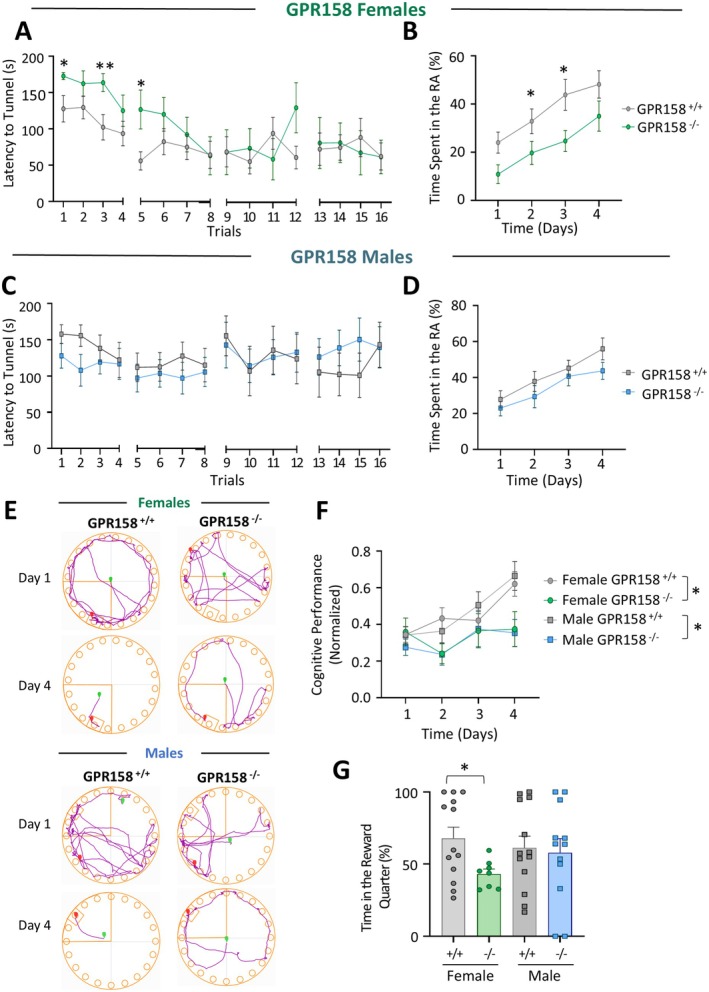
Barnes Maze performance in female and male *GPR158* ^*+/+*^ and *GPR158* ^*−/−*^ mice. (A) The latency to enter the reward tunnel over 4 days for female *GPR158* ^*+/+*^ and *GPR158* ^*−/−*^ mice (*N* = 8–14/genotype). (B) The percentage of time spent in the reward area (RA) immediately surrounding the reward tunnel for female *GPR158* ^*+/+*^ and *GPR158* ^*−/−*^ mice. (C) The latency to enter the reward tunnel over 4 days for male *GPR158* ^*+/+*^ and *GPR158* ^*−/−*^ mice (*N* = 11–14/genotype). (D) The percentage of time spent in the reward area (RA) immediately surrounding the reward tunnel for male *GPR158* ^*+/+*^ and *GPR158* ^*−/−*^ mice. (E) Representative traces of Day 1 and Day 4 of acquisition training for *GPR158* ^*+/+*^ and *GPR158* ^*−/−*^ mice. (F) Change in cognitive performance towards the development of spatial strategies during the acquisition training period. (G) The percentage of time spent in the reward quarter during the probe trials. Data shown as means ± SEM; *p < 0.05, **p < 0.01.

We subjected female and male GPR158^−/−^ and GPR158^+/+^ mice to the Barnes Maze test to assess spatial learning and memory. During the 16 acquisition trials (with 4 trials/day), mice learned to locate and enter the escape tunnel located below the target hole on the platform. During acquisition training, female mice showed a significant effect over the trial sessions, indicating that latencies decreased over training sessions (Figure 1A; trial F_(15, 300)_ = 11.65, *p* < 0.0001). However, female GPR158^−/−^ mice were found to have a longer latency to enter the escape tunnel during the initial trials than GPR158^+/+^ mice (genotype *F*_(1, 20)_ = 1.280, *p* = 0.2714; trial × genotype *F*_(15, 300)_ = 1.865, *p* < 0.05). Female GPR158^−/−^ mice spent significantly less time in the reward area compared to GPR158^+/+^ mice (Figure 1B; day *F*_(3, 60)_ = 18.59, *p* < 0.0001; genotype *F*_(1, 20)_ = 4.383, *p* < 0.05; day × genotype *F*_(3, 60)_ = 0.3926, *p* = 0.758). In contrast, male GPR158^+/+^ and GPR158^−/−^ mice showed similar latency times during the acquisition trials of the Barnes Maze (Figure 1C; day *F*_(5, 315)_ = 3.817, *p* < 0.0001; genotype *F*_(1, 21)_ = 0.07028, *p* = 0.79; day × genotype *F*_(15, 315)_ = 1.333, *p* = 0.1807) and time spent in the reward area (Figure 1D; day *F*_(3, 69)_ = 16.64, *p* < 0.0001; genotype *F*_(1, 23)_ = 1.586, *p* = 0.2205; day × genotype *F*_(3, 69)_ = 0.4807, *p* = 0.697). Over this acquisition period, the traces revealed that GPR158^+/+^ and GPR158^−/−^ mice used different strategies to find the escape tunnel (Figure 1E), and we therefore analyzed the cognitive performance by identifying whether mice learned to use a spatial strategy approach comparing both male and female cohorts. Over the acquisition period, the traces revealed that male and female GPR158^+/+^ mice showed the development of spatial strategies with increasing cognitive performance, whereas female and male GPR158^−/−^ mice's performance did not change over the four‐day period (Figure 1F; day *F*_(3, 723)_ = 95.63, *p* < 0.0001; genotype *F*_(1, 45)_ = 6.68, *p* = 0.013; sex *F*_(1, 45)_ = 0.04, *p* = 0.84; sex × genotype interaction *F*_(1, 45)_ = 2.80, *p* = 0.100). During the probe trial, the escape tunnel was removed and the time spent in the reward quarter was calculated. We found that female GPR158^−/−^ mice spent significantly less time in the reward quarter compared to female GPR158^+/+^ mice, whereas there was no difference between male GPR158^+/+^ and GPR158^−/−^ mice (Figure 1G; genotype *F*_(1, 45)_ = 7.94, *p* = 0.007; sex *F*_(1, 45)_ = 1.54, *p* = 0.022; genotype × sex *F*_(1, 45)_ = 2.97, *p* = 0.0914).

**FIGURE 2:**
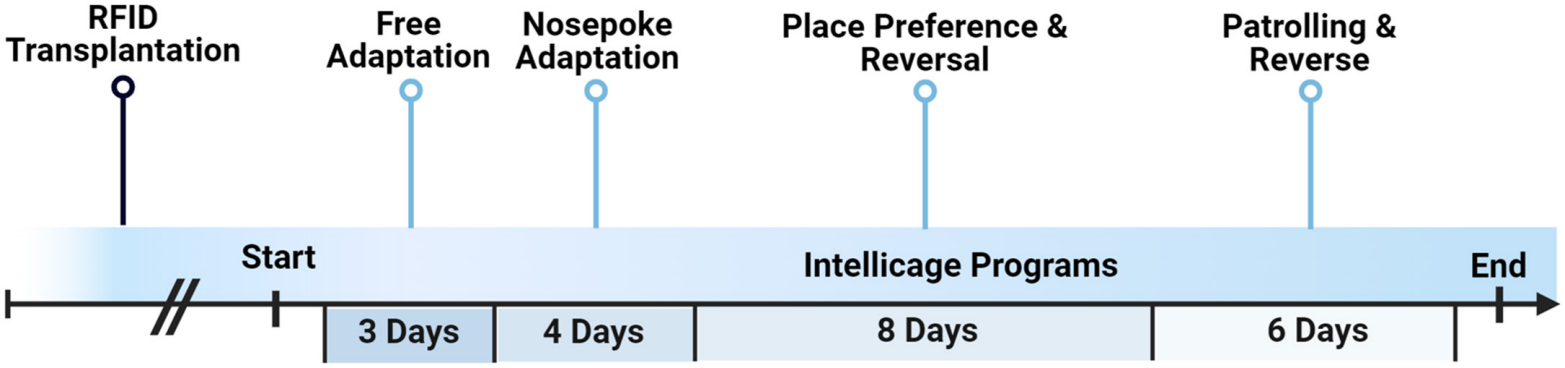
Timeline of experimental procedures in the IntelliCage. Male and female *GPR158* ^*+/+*^ and *GPR158* ^*−/−*^ mice were subcutaneously injected with RFID chips 1 week before starting behavioral paradigms: Free Adaptation, Nosepoke Adaptation, Place Preference and Reversal, and Patrolling and Reverse.

**FIGURE 3:**
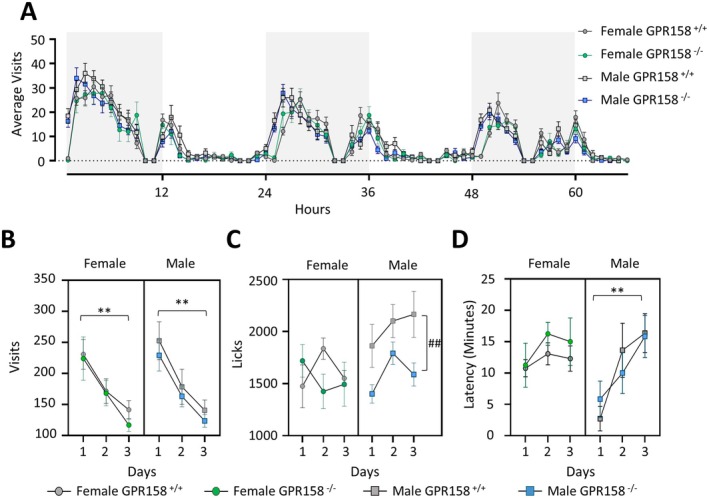
Free Adaptation in female and male *GPR158* ^*+/+*^ *and GPR158* ^*−/−*^ mice. (A) Number of visits to corners over 3 light and dark cycles in female and male mice. Grey blocks indicate the dark cycle. (B) The number of corners visits per day for female (*N* = 8–13/genotype) and male (*N* = 11–12/genotype) *GPR158* ^*+/+*^ *and GPR158* ^*−/−*^ mice. (C) The average number of licks per day. (D) The average latency to make the first visit at any corner per day. Data shown as means ± SEM; **p < 0.01 versus Day 1, ^##^ *p* < 0.01 versus wildtype.

We monitored activity from group‐housed GPR158^−/−^ and GPR158^+/+^ mice using IntelliCages under a schedule of free adaptation, nose‐poke adaptation, place learning and reversal followed by a patrolling paradigm (Figure 2). Activity was recorded for each mouse by a RFID transponder. Initially, we assessed the free adaptation period where mice could move freely for 3 days and had access to all 8 water bottles. As expected, peaks of activity were observed during the dark periods of female and male GPR158^−/−^ and GPR158^+/+^ mice with minimal activity occurring during the light periods (Figure 3A). We observed that the number of corner visits decreased over the days for each group, showing habituation to their new environment regardless of sex or genotype (Figure 3B; day *F*_(2, 80)_ = 58.06, *p* < 0.0001, genotype, *F*_(1, 40)_ = 0.6092, *p* = 0.4397; sex *F*_(1, 40)_ = 0.091, *p* = 0.765; sex × genotype *F*_(1, 40)_ = 0.032, *p* = 0.8578). The total number of licks over this period did not change with no effect of sex but a significant effect of genotype was identified (Figure 3C; day *F*_(2, 80)_ = 2.485, *p* = 0.0897; sex *F*_(1, 40)_ = 3.010, *p* = 0.09; genotype *F*_(1, 40)_ = 4.517, *p* = 0.0398). Further comparison revealed that male GPR158^+/+^ mice had a significantly higher number of licks than male GPR158^−/−^ mice (*p* < 0.0045). For the latency to visit the first corner, there is a significant effect of time but not an effect of genotype or sex (Figure 3D, day *F*_(2, 80)_ = 7.087, *p* < 0.001; genotype *F*_(1, 40)_ = 0.269, *p* = 0.607; sex *F*_(1, 40)_ = 1.958, *p* = 0.169; sex × genotype *F*_(1, 40)_ = 0.5283, *p* = 0.4716). Overall, the data from the free adaptation phase show that both GPR158^+/+^ and GPR158^−/−^ mice habituate to the new environment over the 3‐day exposure.

**FIGURE 4:**
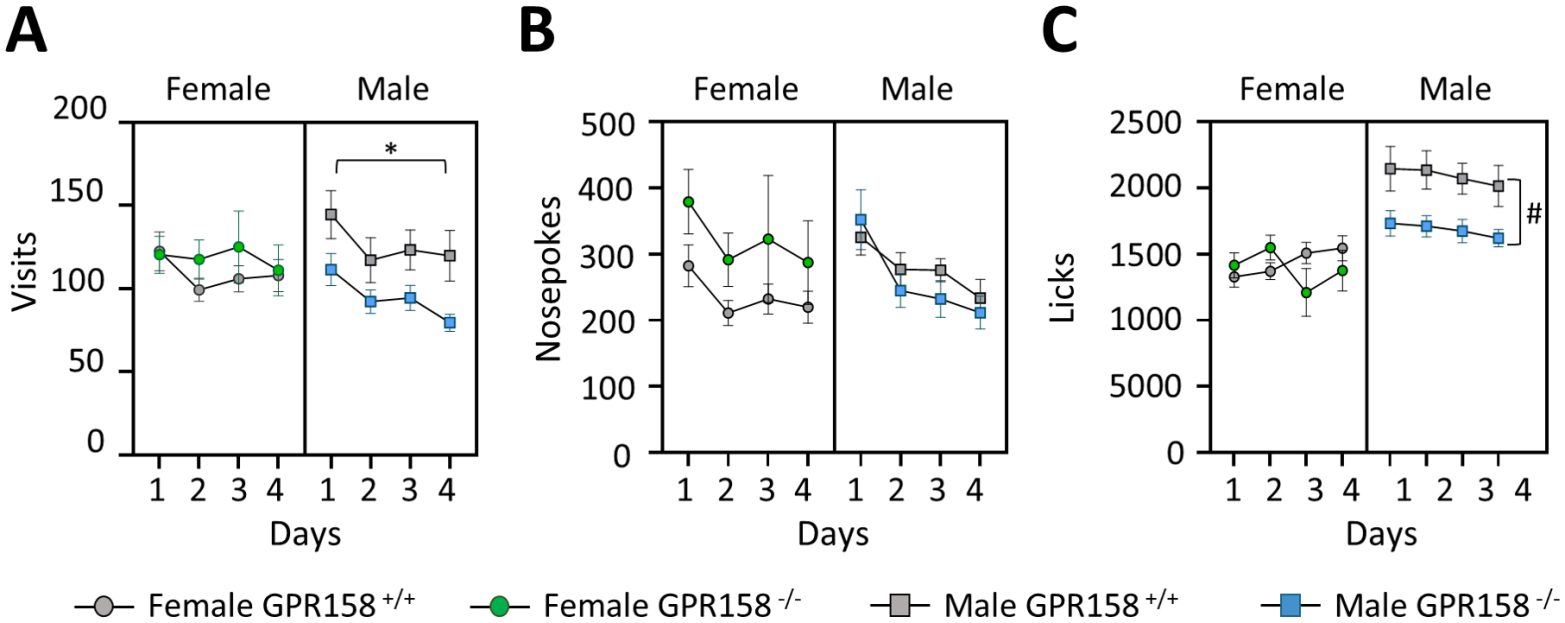
Nosepoke Adaptation in female and male *GPR158* ^*+/+*^ and *GPR158* ^*−/−*^ mice. (A) Average number of visits for female (*N* = 8–13/genotype) and male (*N* = 11–12/genotype) *GPR158* ^*+/+*^ *and GPR158* ^*−/−*^ mice. (B) The number of nosepokes made per day. (C) The number of licks performed over 4 days. Data shown as means ± SEM; *p < 0.05 versus Day 1, ^#^ *p* < 0.01, versus wildtype.

Following the free adaptation phase, mice were exposed to 4 days of nose‐poke adaptation where mice learned to perform a nose‐poke to gain access to the water bottles. For the number of visits, there was a significant effect of day with no effect of sex or genotype (Figure 4A; day *F*_(3, 120)_ = 9.181, *p* < 0.001; genotype *F*_(1, 40)_ = 1.196, *p* = 0.2806; sex *F*_(1, 40)_ = 0.1129, *p* = 0.7386; sex × genotype *F*_(1, 40)_ = 4.155, *p* = 0.0482). There is a decrease in the nose‐pokes over time with no effect of sex or genotype as each group decreased the number of nose‐pokes during these sessions (Figure 4B; day *F*_(3, 120)_ = 12.40, *p* < 0.0001; genotype *F*_(1, 40)_ = 1.557, *p* = 0.2194; sex *F*_(1, 40)_ = 0.2442, *p* = 0.623). Across the 4‐day session, lick counts did not vary significantly with day (Figure 4C; day *F*_(3, 120)_ = 0.8278, *p* = 0.481) but there were robust main effects of sex and genotype (sex *F*_(1,40)_ = 28.53, *p* < 0.001, genotype *F*_(1, 40)_ = 6.614, *p* = 0.013). Post hoc comparison showed that male GPR158^+/+^ mice exhibited a higher number of licks than male GPR158^−/−^, indicating that the genotype effect on licking behavior was driven largely by increased licking in wildtype males. Altogether, both GPR158^+/+^ and GPR158^−/−^ learned to nose‐poke to gain access to water.

**FIGURE 5:**
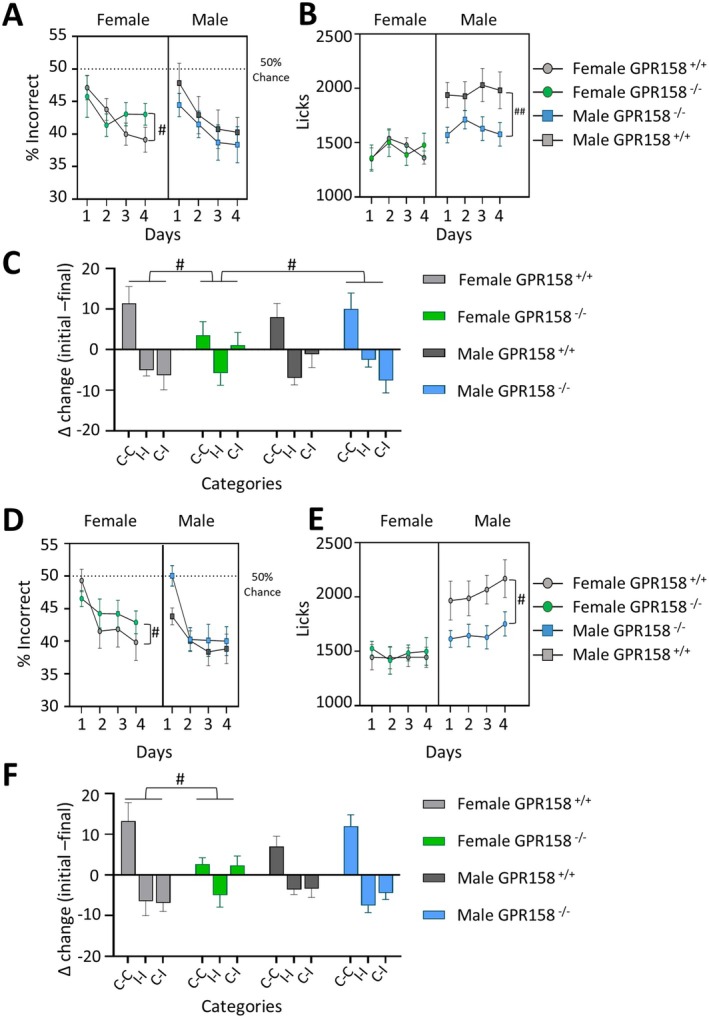
Place preference and reversal in both sexes of *GPR158* ^*+/+*^ and *GPR158* ^*−/−*^ mice. (A) Place error shown as percent incorrect over time for place preference for female (*N* = 8–13/genotype) and male (*N* = 11–12/genotype) *GPR158* ^*+/+*^ *and GPR158* ^*−/−*^ mice. (B) The number of licks at the correct corners over time for place preference. (C) Delta (Δ) change in the proportion of return across the behavioral categories; Correct–Correct (C–C), Incorrect–Incorrect (I–I), and Correct–Incorrect (C–I) over place preference learning in females and males. (D) Place error over time for reversal learning in *GPR158* ^*+/+*^ *and GPR158* ^*−/−*^ mice. (E) The number of licks at the correct corners over time for place reversal. (F) Δ Change in the proportion of return to C–C, I–I, and C–I, over place reversal learning. Data shown as means ± SEM; ^#^ *p* < 0.05, ^##^ *p* < 0.01.

Next, we tested the mice in a place learning paradigm where mice are assigned access to two corners (correct corners) in which they could access water in response to a consecutive 5 s nose‐poke. The other corners are accessible for a mouse to visit but will not have access to the bottles (incorrect corners). The place error was analyzed using a three‐way repeated‐measures ANOVA with days as a within‐subject factor and sex and genotype between‐subject factors. There was a significant main effect of day (*F*_(3, 120)_ = 18.72, *p* < 0.001) indicating that error rates changed across sessions (Figure 5A). The main effects of sex and genotype were not significant (sex *F*_(1,40)_ = 1.09, *p* < 0.30; genotype *F*_(1, 40)_ = 0.54, *p* = 0.47) but a significant sex × genotype interaction was detected (*F*_(1,40)_ = 4.37, *p* = 0.043). Comparison within females showed a difference between the genotypes (*p* < 0.05), where the incorrect response did not change over time for GPR158^−/−^ mice but a decrease was observed for GPR158^+/+^ mice. Both male GPR158^+/+^ and GPR158^−/−^ displayed a decrease in the incorrect responses over time. The total number of licks over this period did not change (Figure 5B; *F*_(3, 120)_ = 2.426, *p* = 0.07). While there was no effect of sex, a significant effect of genotype was identified (sex *F*_(1, 40)_ = 13.95, *p* = 0.0006; genotype *F*_(1, 40)_ = 3.070, *p* = 0.0874). Further comparison revealed that male GPR158^+/+^ mice had a significantly higher number of licks during place preference than GPR158^−/−^ mice (*p* = 0.0045). We analyzed the behavioral transition categories of correct–correct (C–C) nose‐pokes over incorrect–incorrect (I–I) or incorrect–correct/correct–incorrect (C–I) nose‐pokes during initial and final days (Figure 5C). The ART repeated‐measures analysis revealed a highly significant behavioral transition category (*F*_(2, 216, 3)_ = 205.01, *p* < 2.2 × 10^−16^), indicating that the mean numbers of transitions differed significantly among the three behavioral transition categories (C–C, C–I, and I–I). During the initial phase, mice exhibited the greatest mean number of correct‐to‐incorrect (C–I) transitions (mean = 64.2), followed by consecutive correct (C–C) transitions (mean = 27.2) and consecutive incorrect (I–I) transitions (mean = 20.7). A significant behavioral transition category × phase interaction was also detected (*F*_(2, 216, 3)_ = 7.13, *p* = 0.001), indicating that the pattern of behavioral transitions changed between the initial and final phases. Specifically, the mean number of consecutive correct transitions (C–C) increased from 27.2 during the initial phase to 34.6 during the final phase (7.4 transitions), whereas correct‐to‐incorrect transitions (C–I) decreased from 64.2 to 55.8 (−8.4 transitions) and consecutive incorrect transitions (I–I) decreased from 20.7 to 14.3 (−6.4 transitions). Together, these changes indicate a shift toward more consistent correct spatial choices over time. No significant main effects of sex, genotype, or phase were detected. However, a significant sex × genotype interaction was observed (*F*_(1, 169, 5)_ = 4.77, *p* = 0.030), indicating that the influence of genotype differed between males and females. To identify the source of this interaction, we performed post hoc comparisons using estimated marginal means from the ART model to compare genotypes within each sex and sexes within each genotype. These analyses showed that the overall pattern of behavioral transitions differed significantly between female GPR158^−/−^ and GPR158^+/+^ mice (*p* = 0.024), whereas no genotype difference was detected among males (*p* = 0.366). Similarly, the overall pattern of behavioral transitions differed significantly between female and male GPR158^−/−^ mice (*p* = 0.008), whereas no significant sex difference was observed among GPR158^+/+^ mice (*p* = 0.444). Although the sex × genotype × behavioral transition category interaction approached significance (*p* = 0.064), neither this nor any higher‐order interaction involving phase reached statistical significance.

The reverse period immediately followed where the water bottles that were originally accessible were inaccessible, and the inaccessible corners were accessible. We analyzed the place error and found a main effect of day (Figure 5D; *F*_(3,120)_ = 14.97, *p* < 0.0001), indicating a change in performance across days. There were no significant effects of sex or genotype. Comparison of females revealed a significant interaction of time x genotype (*p* < 0.05) in which the percentage of incorrect responses did not change over days for GPR158^−/−^ mice but GPR158^+/+^ mice had fewer incorrect responses during the reversal period. There was no significant difference in the percentage of incorrect responses between male GPR158^+/+^ and GPR158^−/−^ mice as both groups exhibited a decrease in the number of incorrect responses (*p* > 0.05). The total number of licks over this period did not change but a significant effect of sex was found (Figure 5E, day *F*_(3, 120)_ = 1.99, *p* = 0.1191; sex *F*_(1, 40)_ = 12.60, *p* = 0.001). Male GPR158^+/+^ mice had significantly more licks than GPR158^−/−^ mice (*p* < 0.05). Next, we assessed the change in responses during the initial and final phase of the reversal sessions (Figure 5F). The ART repeated‐measures analysis revealed a highly significant main effect of behavioral transition category (*F*_(2, 216, 2)_ = 267.66, *p* < 2.2 × 10^−16^), indicating that the mean numbers of transitions differed significantly among the three behavioral transition categories (C–C, C–I, and I–I). A significant behavioral transition category × phase interaction was also detected (*F*_(2,216,3)_ = 11.21, *p* = 2.34 × 10^−5^), indicating that the pattern of behavioral transitions changed between the initial and final phases. Specifically, the mean number of C–C increased from 27.4 during the initial phase to 34.6 during the final phase (7.2 transitions), whereas C–I decreased from 65.1 to 59.6 (−5.5 transitions) and I‐I decreased from 22.8 to 15.7 (−7.1 transitions). Together, these changes indicate a shift toward more consistent correct spatial choices over time. Although there were no significant main effects of sex, genotype, or phase, there were significant sex × genotype (*F*_(1, 172, 4)_ = 8.22, *p* = 0.0047) and sex × genotype × category (*F*_(2, 216, 3)_ = 4.47, *p* = 0.0125) interactions. Post hoc comparisons comparing genotypes separately showed that the transitions differed between female GPR158^−/−^ and GPR158^+/+^ mice (*p* < 0.05) with wildtype mice exhibiting more C–C responses than knockout counterparts.

**FIGURE 6:**
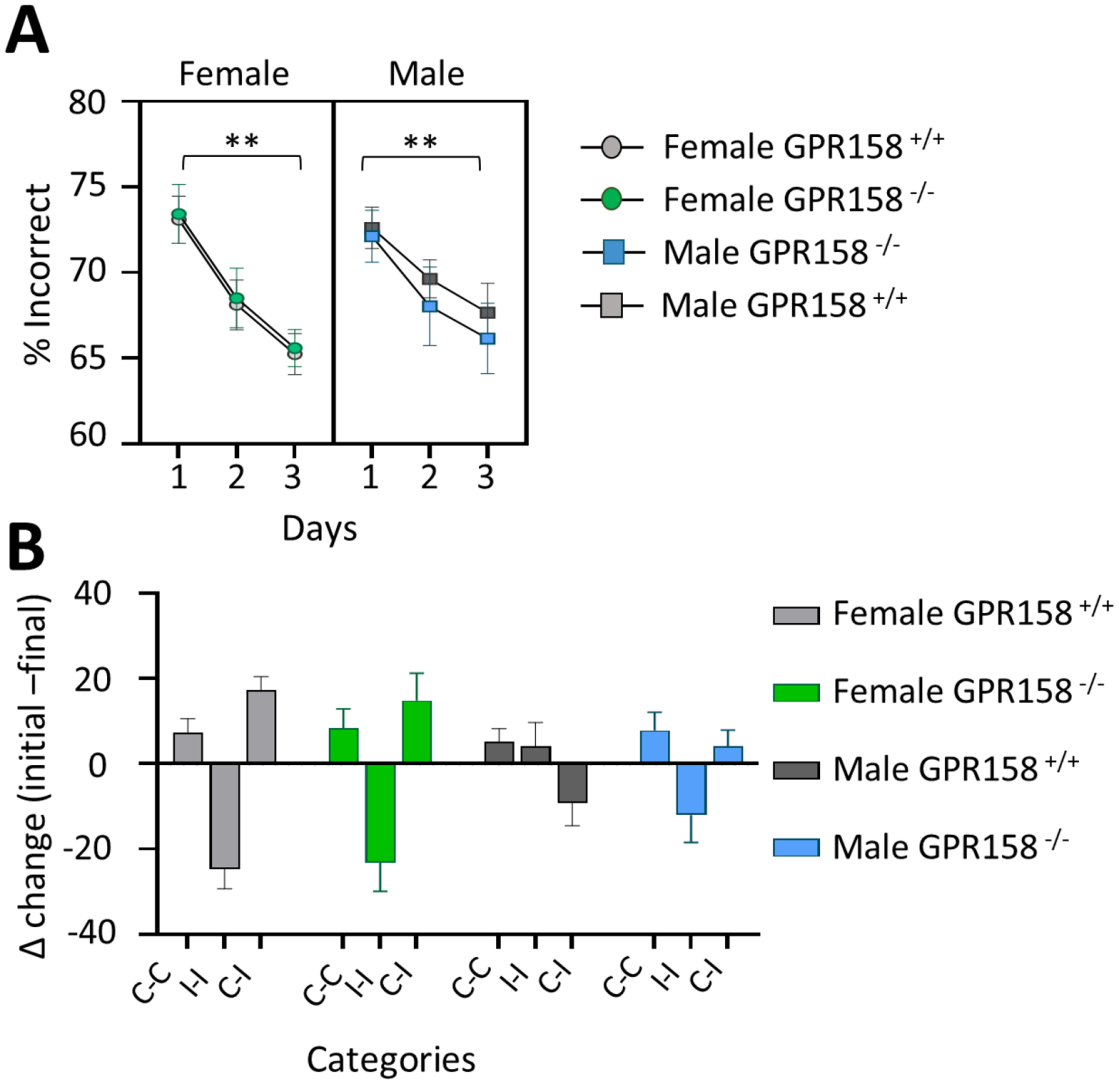
Assessment of female and male *GPR158* ^*+/+*^ and *GPR158* ^*−/−*^ in a patrolling paradigm. (A) Change in place error during patrolling for female (*N* = 8–13/genotype) and male (*N* = 11–12/genotype) *GPR158* ^*+/+*^ *and GPR158* ^*−/−*^ mice. (B) Δ change in the proportion of return across the behavioral categories; Correct–Correct (C–C), Incorrect–Incorrect (I–I), and Correct–Incorrect (C–I), patterns of return over patrolling for females and males. Data shown as means ± SEM; **p < 0.01.

A patrolling paradigm was used to evaluate working memory, where the correct corner was programed to switch in a clockwise position with every correct response. Analyzing the place error, we found a significant main effect of time (*F*_(2, 80)_ = 21.38, *p* < 0.001) with no significant effect on sex or genotype (Figure 6A). We analyzed the change in behavioral categories (C–C, C–I, I–I) from Day 1 and Day 3 and found a significant effect in transition categories (Figure 6B; *F*_(2, 216,1)_ = 274.43, *p* < 2.2 × 10^−16^). There was only a slight change in C–C response from a mean of 9.6 to 10.2 (0.6 transitions), whereas C–I decreased from 51 during the initial phase to 44.8 during the final phase (−6.2 transitions), and I‐I response also decreased from a mean of 63.4 to 44.4 (−19 transitions). Post hoc comparison showed that each group's incorrect responses decreased over time (*p* < 0.05). Together, these results indicate a reduction in incorrect transition behaviors over the patrolling protocol.

## 4 Discussion

In our study we show that GPR158 knockout mice display deficits in spatial learning and cognitive flexibility using both standard and automated phenotyping paradigms. In the Barnes Maze test, both male and female GPR158^−/−^ mice showed deficits in the spatial strategies, with female GPR158^−/−^ showing more pronounced deficits as their latency to the tunnel, time spent in the RA, and time in reward quarters all showed deficits. Using an automated homecage system, we conducted a longitudinal study of group‐housed mice to monitor four different behavioral phases all in their homecage environment; free adaptation, nose‐poke adaptation, place preference, and patrolling. During the free adaptation period, GPR158^+/+^ and GPR158^−/−^ exhibit similar levels of habituation and exploratory behavior in both male and female cohorts. There was also no difference between genotypes during the nose‐poke adaptation period. In the place preference test, we found that only female GPR158^−/−^ mice showed impaired spatial learning. Female GPR158^+/+^ mice displayed significantly fewer incorrect responses over both the preference and reversal periods, whereas GPR158^−/−^ mice showed no change in the number of incorrect responses across these phases. In contrast, both male GPR158^+/+^ and GPR158^−/−^ mice exhibit comparable place preference learning with a decreasing percentage of incorrect responses over time. Overall, female GPR158^−/−^ mice showed deficits in spatial learning regardless of whether testing occurs in isolation or a group environment.

Several studies have shown that GPR158^−/−^ mice show deficits in learning and memory paradigms as assessed with standard behavioral methods, including the Morris Water Maze and a traditional Barnes Maze [8, 24]. In addition, GPR158^−/−^ mice show deficits in object recognition, displaying difficulty distinguishing between novel and familiar objects in the novel object recognition test, as well as aberrant responses in the passive avoidance task [6]. Many of these studies have been conducted in only one sex, and comparative analysis between males and females has not been performed. While we found deficits in the spatial strategies that were used in the Barnes Maze for both male and female GPR158^−/−^ mice, only female knockout mice showed deficits in the place learning and reversal in the IntelliCage. This describes a sex difference between males and females GPR158^−/−^ mice towards spatial learning when testing in a group environment. Spatial learning tasks in the IntelliCage provide a less stressful testing environment with social enrichment [25]. Mice maintained under an enriched environment have shown increased performance of hippocampus‐dependent spatial working memory tasks [26]. Environmentally enriched mice showed enhanced spatial learning and reversal learning in the Morris Water Maze task, as well as enhanced LTP in the hippocampal dentate gyrus compared to reductions found in socially isolated mice [15]. Furthermore, in group testing environments, mice have the potential to use social observation and perseveration behaviors to make spatial choices. This has been observed with aging female mice, where mice used non‐spatial strategies to find a reward (drinking) corner in the IntelliCage [27]. These findings suggest that male GPR158^−/−^ mice may have compensated for spatial learning deficits by adopting alternative, nonspatial behavioral strategies during place learning and reversal in the IntelliCage.

The effect of GPR158 signaling on learning has been speculated to be due to its role in the structural organization and formation of the synapse. Loss of GPR158 affects the hippocampal architecture and synaptic organization as it is a pathway‐specific organizer and has been shown to mediate hippocampal‐dependent learning [7, 8]. It has been shown that GPR158^−/−^ mice have reductions in dendrite architecture, complexity, and electrophysiological properties and negatively affects hippocampal‐dependent learning in a Morris water maze [6, 8]. Similar to our observed deficits in spatial learning tasks, these deficits in spatial learning are likely contributed to by aberrant neurocircuitry in the hippocampus of the GPR158^−/−^ mice.

From a clinical perspective, GPR158 has been associated with Alzheimer's disease with studies demonstrating disruptive GPR158 levels in both the cortex of patients with Alzheimer's disease and animal models [28, 29, 30]. While these studies have shown an association, it has been difficult to investigate the signaling mechanism until recently as GPR158 has been classified as an orphan receptor. Glycine has been proposed to be the endogenous ligand for GPR158, demonstrating it as the metabotropic glycine receptor for this amino acid [31]. Activation of GPR158 by glycine has been shown to enhance the intrinsic excitability of medium spiny neurons in the striatum and in cortical neurons [31, 32] Besides glycine, osteocalcin, a bone‐derived hormone, has also been proposed as an endogenous ligand [8]. Interestingly, reduced levels of osteocalcin have been documented in cerebrospinal fluid of Alzheimer's disease patients, and in the aging population associated with cognitive deterioration, as well as mice deficient in osteocalcin exhibited impaired spatial learning and memory dependent on the hippocampus [33, 34, 35] Understanding how GPR158 is activated by these specific ligands and how it modulates spatial learning requires further exploration.

## 5 Conclusions

Together this study has demonstrated the value of an automated group home cage to characterize mouse models whose behavioral output may be sensitive to stress, environmental conditions, and social behaviors. Standard behavioral testing may induce multiple confounds and is only able to provide snapshots of individual behavior in a novel, unfamiliar environment. This longitudinal study minimizing these confounds in a social setting is necessary to illuminate a more naturalized role of GPR158 in cognition and may provide valuable information on the impact of confounds induced by traditional behavioral testing methods. By mitigating stress and social isolation during behavioral testing we have shown that the spatial deficits observed in female GPR158^−/−^ mice are due to cognitive deficits.

## Funding

This work was supported by a startup fund from the University of Maryland Baltimore County.

## Conflicts of Interest

The authors declare no conflicts of interest.

## Data Availability

The data that support the findings of this study are available from the corresponding author upon reasonable request.
